# SCF^β-TRCP^ E3 ubiquitin ligase targets the tumor suppressor ZNRF3 for ubiquitination and degradation

**DOI:** 10.1007/s13238-018-0510-2

**Published:** 2018-03-01

**Authors:** Yanpeng Ci, Xiaoning Li, Maorong Chen, Jiateng Zhong, Brian J. North, Hiroyuki Inuzuka, Xi He, Yu Li, Jianping Guo, Xiangpeng Dai

**Affiliations:** 1 School of Life Science and Technology, Harbin Institute of Technology, Harbin, 150001 China; 2 Department of Pathology, Beth Israel Deaconess Medical Center, Harvard Medical School, Boston, MA 02215 USA; 3 Department of Biochemistry and Molecular Biology, Shanxi Medical University, Taiyuan, 030001 China; 4 The FM Kirby Neurobiology Center, Children’s Hospital Boston, Harvard Medical School, Boston, MA 02115 USA; 5 Department of Oncology, The First Affiliated Hospital of Xinxiang Medical University, Xinxiang, 453100 China; 6 Center for Advanced Stem Cell and Regenerative Research, Tohoku University Graduate School of Dentistry, Sendai, 980-8575 Japan

**Keywords:** ZNRF3, β-TRCP, Wnt, ubiquitination, CKI

## Abstract

Wnt signaling has emerged as a major regulator of tissue development by governing the self-renewal and maintenance of stem cells in most tissue types. As a key upstream regulator of the Wnt pathway, the transmembrane E3 ligase ZNRF3 has recently been established to play a role in negative regulation of Wnt signaling by targeting Frizzled (FZD) receptor for ubiquitination and degradation. However, the upstream regulation of ZNRF3, in particular the turnover of ZNRF3, is still unclear. Here we report that ZNRF3 is accumulated in the presence of proteasome inhibitor treatment independent of its E3-ubiquitin ligase activity. Furthermore, the Cullin 1-specific SCF complex containing β-TRCP has been identified to directly interact with and ubiquitinate ZNRF3 thereby regulating its protein stability. Similar with the degradation of β-catenin by β-TRCP, ZNRF3 is ubiquitinated by β-TRCP in both CKI-phosphorylation- and degron-dependent manners. Thus, our findings not only identify a novel substrate for β-TRCP oncogenic regulation, but also highlight the dual regulation of Wnt signaling by β-TRCP in a context-dependent manner where β-TRCP negatively regulates Wnt signaling by targeting β-catenin, and positively regulates Wnt signaling by targeting ZNRF3.

**Electronic supplementary material:**

The online version of this article (10.1007/s13238-018-0510-2) contains supplementary material, which is available to authorized users.

## Introduction

Wnt signaling, discovered three decades ago, has been tightly linked with fundamental growth control and tissue development (Nusse and Varmus, 1982; Goldstein et al., 2006; Clevers, 2006). The well-characterized canonical Wnt/β-catenin pathway is triggered by the interaction of Wnt ligands with a receptor complex (FZD and LRP5/6), which in turn results in the accumulation of β-catenin to positively promote transcription of a cohort of Wnt targeted genes (Nusse and Clevers, 2017; MacDonald and He, 2012; Peifer and Polakis, 2000; Hart et al., 1999). As the primary Wnt effector, the transcriptional activator β-catenin targets genes largely regulating normal tissues stem cell progression and is involved in tissue development and tissue boundary control (Goldstein et al., 2006; Clevers, 2006; Espada et al., 2009). More interestingly, the activation of β-catenin has also been involved in T reg cell exhaust or tumor cell evasion from immune surveillance by targeting PD1 and PDL1, respectively (Spranger et al., 2015; Yang et al., 2017). On the other hand, Wnt targeted genes, including *ZNRF3* and *AXIN2* provide negative feedback mechanisms upon the Wnt pathway (Hao et al., 2012; Lustig et al., 2002). Due to the critical role of Wnt signaling in cell growth and normal stem cell progression, genetic alteration of the components within this pathway including the lost-of-function mutations/deletions of *APC*, *LRP5*, *AXIN2* promotes different diseases, including bone density defects (*LRP5*, *Wnt1*) (Van Wesenbeeck et al., 2003), familial exudative vitreoretinopathy (*FZD4*) (Toomes et al., 2004) and colon cancer (*APC*, *AXIN2*) (Morin et al., 1997; Lammi et al., 2004). Thus, understanding the upstream regulation of the Wnt signaling pathway is necessary for developing new methods to alleviate these diseases, especially colon cancers.

As a downstream target of Wnt signaling, ZNRF3/RNF43 recently has been identified to negatively regulate the Wnt pathway (Hao et al., 2012; Koo et al., 2012). Biochemically, ZNRF3, as a single trans-membrane E3 ligase, could directly bind and target FZD for ubiquitination and degradation specifically when the Wnt pathway is activated (Hao et al., 2012). Moreover, *ZNRF3*/*RNF43* are frequently mutated in tumors, and depletion of *ZNRF3* contributes to the continuous activation of the Wnt pathway in driving stem cells (Koo et al., 2012). Although R-spondin-mediated endocytosis of ZNRF3 has partially explained the regulation of ZNRF3 (Hao et al., 2012), whether ZNRF3 undergoes auto-ubiquitination or is ubiquitinated by other E3 ligase(s) is not well defined.

Cullin-based E3-ubiquitin ligases make up the largest group ligases in ubiquitin-proteasome systems (UPS) and target distinguished substrates to govern diverse cellular processes including cell cycle progression, cell apoptosis and cell differentiation (Shen et al., 2013; Wang et al., 2014). Among them, the SCF (Skp1/Cullin 1/F-box protein) E3 ligase complex has been extensively studied and plays major roles in regulating various cellular processes including, but not limited to, cell cycle and stem cell regulations (Wang et al., 2014; Skowyra et al., 1997). Based on their biological functions, SCF E3 ligases have been further divided into three groups: oncogenic (SKP2), tumor suppressive (FBW7) and context-dependent (β-TRCP) E3-ubiquitin ligase, in which loss-of-function mutation/deletion of tumor suppressive F-box proteins, such as *FBW7*, have been shown to regulate tumorigenesis (Welcker and Clurman, 2008; Akhoondi et al., 2007).

Unlike tumor suppressive E3 ligase such as FBW7 and FBXO4, or oncogenic E3 ligase such as SKP2, β-TRCP (β-TRCP1 and β-TRCP2) displays context-dependent (tumor type or cellular context) functions in tumorigenesis (Wang et al., 2014; Skowyra et al., 1997). Genetically, *β-TRCP1* knockout mice (*Btrcp1*^−/−^) do not have increased cancer incidence. However, tissue-specific knockout of *β-TRCP1* in mammary glands of female mice displays a hypoplastic phenotype (Nakayama et al., 2003; Guardavaccaro et al., 2003). In contrast, around 40% of MMTV *β-TRCP1* transgenic mice targeting the epithelial tissues could develop tumors including mammary, ovarian and uterine tumors, indicating that β-TRCP1 could promote epithelial tumorigenesis *in vivo* (Kudo et al., 2004). By targeting β-catenin, β-TRCP1 plays a negative role in regulating the Wnt pathway, partially explaining how somatic mutations in *β-TRCP1*/*2* preventing their E3 ligase activity identified in human gastric cancer correlated with stabilization of β-catenin in these tissues and development of tumors (Saitoh and Katoh, 2001). On the other hand, by targeting IκB, β-TRCP1 plays a negative role in regulating the NF-κB pathway, a central regulator of chronic inflammation (Spencer et al., 1999).

Here we report that SCF^β-TRCP^ E3-ubiquitin ligase complex physically interacts with and ubiquitinates ZNRF3 to mediate ZNRF3 proteasome-dependent degradation. Moreover, the regulation of ZNRF3 by β-TRCP is also regulated in a casein kinase I (CKI) phosphorylation- and degron-dependent manner, which highlight the important roles of β-TRCP in regulation of Wnt pathway by targeting β-catenin in Wnt off and ZNRF3 in Wnt on conditions.

## Results

### Cullin 1 governs ZNRF3 turnover in a proteasome-dependent manner

**Figure 1:**
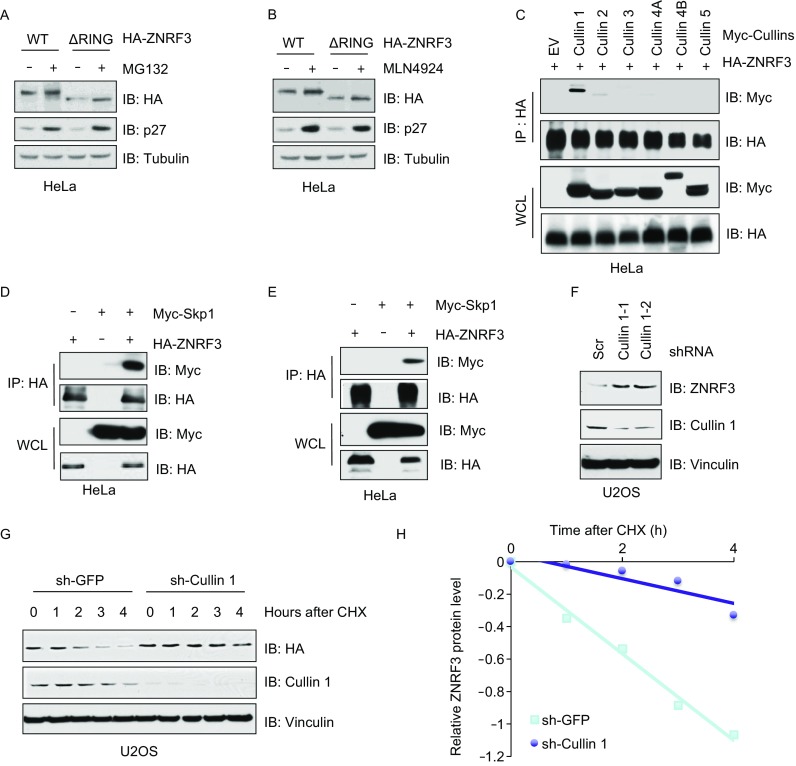
**ZNRF3 protein level is regulated by Cullin 1 based E3 ligases, in a 26S proteasome dependent manner**. (A and B) Immunoblot (IB) analysis of whole cell lysates (WCL) derived from HeLa cells transfected with WT or RING-domain deleted (ΔRING) ZNRF3. Resulting cells were treated with MG132 (10 μmol/L) (A) or MLN4924 (10 μmol/L) (B) for 12 h before harvesting for IB analysis. (C–E) IB analysis of immunoprecipitations (IP) and WCL derived from HeLa cells co-transfected ZNRF3 with various Cullin family proteins (C), Skp1 (D) or Rbx1 (E) after treated with MG132 (10 μmol/L) for 12 h before being harvested for IB analysis. (F) IB analysis of WCL derived from U2OS cells lentivirally infected with control (sh-Scr) or multiple independent shRNAs against *Cullin 1* (sh-Cullin1). Infected cells were selected with 1 μg/mL puromycin for 72 h to eliminate non-infected cells before harvesting. (G and H) IB analysis of WCL derived from the cell lines in (F) treated with CHX (100 mg/L) for different time point (G), relative protein levels were quantified and plotted in (H)

Although a role for ZNRF3 as an E3-ubiquitin ligase has been recently established (Hao et al., 2012; Koo et al., 2012), the regulation, in particular the turnover of ZNRF3, has yet to be investigated. To this end, we treated HeLa cells with the proteasome inhibitor MG132, and observed that the protein level of ZNRF3 was markedly increased (Figs. 1A and S1A). It has been reported that E3 ligases have the ability to undergo auto-ubiquitination and subsequent degradation (de Bie and Ciechanover, 2011; Scaglione et al., 2007). To investigate a possible role of self-ubiquitination in the regulation of ZNRF3 protein stability, we generated an E3-ligase inactive form of ZNRF3 (ΔRING). Notably, we observed that treatment of MG132 could accumulate ZNRF3-ΔRING protein abundance similar to wild-type ZNRF3 (Fig. 1A), suggesting that the protein stability of ZNRF3 is regulated by E3 ligase(s) other than itself. Given that Cullin-based E3 ligases are the biggest group of UPS, we treated HeLa cells with Cullin Nedd8-activating enzyme inhibitor MLN4924 (Soucy et al., 2009), which could markedly increase both WT and ΔRING-ZNRF3 protein levels (Figs. 1B and S1B), indicating that ZNRF3 is degraded in a Cullin-dependent fashion.

Cullins, as the E3 ligase scaffolding subunits, includes 6 Cullin members, Cullin 1, Cullin 2, Cullin 3, Cullin 4A, Cullin 4B, Cullin 5 and Cullin 7 (Sarikas et al., 2011). In order to pinpoint the exact Cullin(s) involved in regulation of ZNRF3, we screened a panel of Cullin proteins, and observed that Cullin 1, but not other Cullin proteins, could interact with ZNRF3 (Fig. 1C). Consistent with the previous observation that Skp1/Rbx1/Cullin 1 form an SCF complex, we found the physiological interaction of Skp1 and Rbx1 with ZNRF3 in cells (Fig. 1D and 1E). More importantly, depletion of *Cullin 1* by shRNAs increased endogenous ZNRF3 protein abundance in U2OS cells (Fig. 1F). Furthermore, to determine whether Cullin 1 modulates ZNRF3 at the protein level, we treated both control and *Cullin 1*-depleted U2OS cells with cycloheximide (CHX), an eukaryote protein synthesis inhibitor, and observed that the half-life of ZNRF3 in *Cullin 1*-depleted cells was significantly prolonged compared with control cells (Fig. 1G and 1H), supporting the notion that Cullin 1-based E3 complex governs ZNRF3 protein abundance.

### β-TRCP interacts with and ubiquitinates ZNRF3

**Figure 2:**
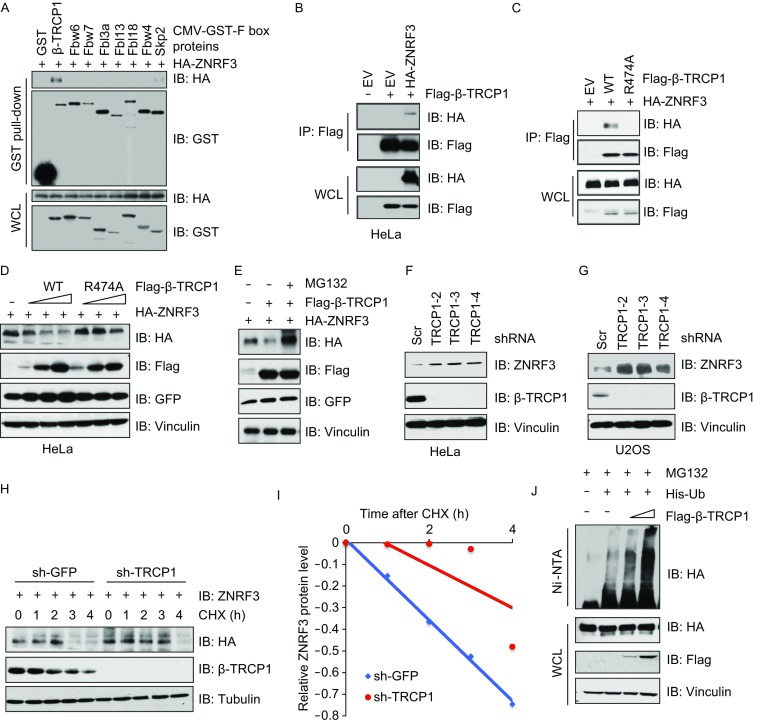
**β-TRCP directly interacts with and promotes the degradation of ZNRF3**. (A–C) IB analysis of IP product and WCL derived from HEK293T (A and C) or HeLa (B) cells transfected with indicated constructs, resulting cells were then treated with MG132 (10 μmol/L) for 12 h before harvesting. (D and E) IB analysis of WCL derived from HeLa cells co-transfected ZNRF3 with different TRCP encoding constructs, treated without (D) or with MG132 (10 μmol/L) (E) for 12 h before harvesting. (F and G) IB analysis of WCL derived from HeLa (F) or U2OS (G) cells lentivirally infected with control (sh-Scr) or independent shRNAs against *β-TRCP1* (sh-TRCP1). Infected cells were selected with 1 μg/mL puromycin for 72 h to eliminate non-infected cells before harvesting. (H and I) IB analysis of WCL derived from cells generating in (F) and treated with CHX (100 mg/L) for different time points (H). The relative protein levels were quantified and plotted in (I). (J) IB analysis of ubiquitin-products and WCL derived from HEK293 cells transfected with indicated constructs. Where indicated Nickel-beads were used to pull down His-tagged Ub proteins

Cullin 1-based SCF complexes contain an F-box protein which serve as the substrate recognition subunit (Wang et al., 2014). In order to identify which F-box proteins are involved in targeting ZNRF3, we performed GST-pulled down assays with a panel of F-box proteins, and observed that β-TRCP1 but not other F-box proteins we examined including FBW7, could physiologically interact with ZNRF3 (Fig. 2A). We further confirmed the interaction of β-TRCP1 with ZNRF3 in cells (Fig. 2B). In addition, we observed the interaction of RNF43, a homolog protein of ZNRF3 which is also a negative regulator of Wnt signaling, with β-TRCP1 (Fig. S1C). To validate the specificity of interaction, we observed that the interaction between β-TRCP1 and ZNRF3, but not RNF43, was impaired when the substrate recognition domain of β-TRCP was mutated to R474A (Figs. 2C and S1D) (Wang et al., 2015). All these results indicate ZNRF3 as a potential ubiquitin substrate of β-TRCP.

We next assayed the effect of β-TRCP on ZNRF3 protein stability and observed that ectopically expressing β-TRCP could efficiently reduce ZNRF3 protein stability in a dose-dependent manner (Fig. 2D). Furthermore, the reduction of ZNRF3 by β-TRCP1 could be restored by treatment with MG132 (Fig. 2E), indicating that the degradation of ZNRF3 mediated by β-TRCP1 largely depended on the proteasome. More interestingly, we observed that depletion of *β-TRCP1* could enhance the basal level of β-catenin, whereas the wnt3a stimulation could enhance Wnt response in *β-TRCP1*-depletion cells compared with control cells partially dependent on the negative regulation of ZNRF3 (Fig. S1F and S1G). Consistent with finding that R474A-β-TRCP1 did not interact with ZNRF3, we also observed that WT-β-TRCP1, but not R474A-β-TRCP1, could degrade ZNRF3 in a dose-dependent manner (Fig. 2D). Moreover, depletion of *β-TRCP1* or *β-TRCP2* respectively or in combination by independent shRNAs could efficiently increase ZNRF3 protein levels (Figs. 2F, 2G and S1E). Depletion of *β-TRCP* could also prolong ZNRF3 protein half-life (Fig. 2H and 2I). To further investigate whether β-TRCP functions as a ZNRF3 E3 ubiquitin ligase, ZNRF3-ΔRING was co-transfected with/without β-TRCP1, and the ubiquitination status of ZNRF3 was significantly increased in the presence of β-TRCP1 (Fig. 2J), indicating β-TRCP as the *bona fide* ZNRF3 upstream E3-ubiquitin ligase.

### CKI promotes β-TRCP-mediated ZNRF3 ubiquitination and degradation

**Figure 3:**
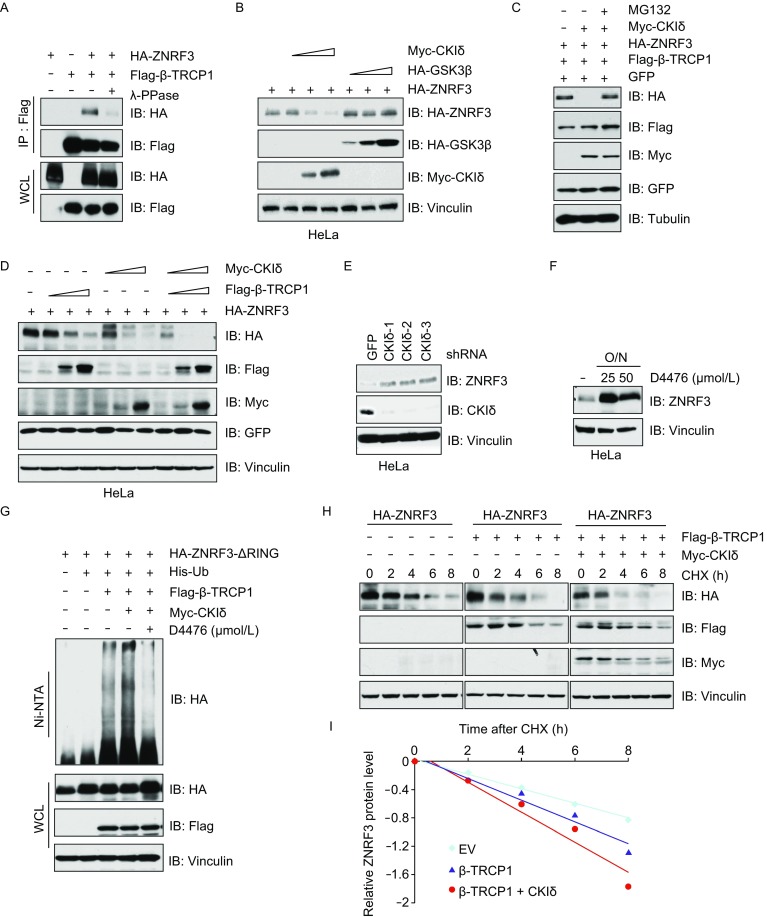
**CKI synergizes with β-TRCP to mediate ZNRF3 degradation**. (A–D) IB analysis of IP and WCL derived from HEK293 (A and C) or HeLa (B and D) cells transfected with indicated constructs, cell lysates were treated with or without phosphatase (PPase) for 30 min (A) or cells were treated with MG132 for 12 h (C) before harvesting. (E) IB analysis of WCL derived from HeLa cells infected with lentivirus for control (sh-Scr) or multiple independent shRNAs against CKIδ (sh-CKIδ). Infected cells were selected with 1 μg/mL puromycin for 72 h to eliminate non-infected cells before harvesting. (F) IB analysis of WCL derived from HeLa cells treated with different concentrations of CKI inhibitor D4476 overnight before harvesting. (G) IB analysis of ubiquitin-products and WCL derived from HEK293 cells transfected with indicated constructs treated with or without CKI inhibitors. Where indicated Nickel-beads were used to pull down His-tagged Ub proteins. (H–I) IB analysis of WCL derived from HeLa cells transfected with indicated constructs and treated with CHX (100 mg/L) for indicated time points (H), the relative protein levels were quantified and plotted in (I)

In most instances, β-TRCP recognizes substrates with phosphorylated Serine residues within a degron motif (DSGxxS) (Shimizu et al., 2017). To validate whether β-TRCP mediates ZNRF3 degradation in a phosphorylation dependent manner, cells were transfected with ZNRF3 and β-TRCP and subsequently treated with or without λ-phosphatase (λ-PPase). We observed that blocking phosphorylation could largely abolish the interaction of β-TRCP with ZNRF3 (Fig. 3A). To identify the upstream kinase regulating ZNRF3 phosphorylation, kinases such as GSK3β, CKI and CKII, which are commonly involved in β-TRCP substrate phosphorylation were assessed. Notably, CKI but not any other kinase examined could decrease ZNRF3 protein level in the presence of β-TRCP (Figs. 3B and S2A), which could be rescued by proteasome inhibitor MG132 treatment (Fig. 3C). Furthermore, we found that the δ variant, a lesser extent of α and ɛ variants, of CKI was involved in degrading ZNRF3 (Fig. S2A). As expected, CKIδ could strongly enhance β-TRCP functions to degrade ZNRF3 compared with expression of CKI or β-TRCP alone (Fig. 3C and 3D).

Next, we found that depletion of *CKIδ* could markedly increase ZNRF3 abundance in HeLa cells (Fig. 3E). Treatment with the CKI inhibitor (D4476) upregulated ZNRF3 protein levels in a dose-dependent manner (Fig. 3F). In addition, CKI could enhance the ability of β-TRCP to ubiquitinate ZNRF3 in cells, which was impaired by treatment with CKI inhibitor D4476 (Fig. 3G). CKIδ could also significantly enhance β-TRCP functions to accelerate the degradation of ZNRF3 (Fig. 3H and 3I). These findings altogether suggest that CKI kinase could facilitate β-TRCP-mediated ZNRF3 ubiquitination and degradation.

### β-TRCP promotes the degradation of ZNRF3 in a degron-dependent manner

**Figure 4:**
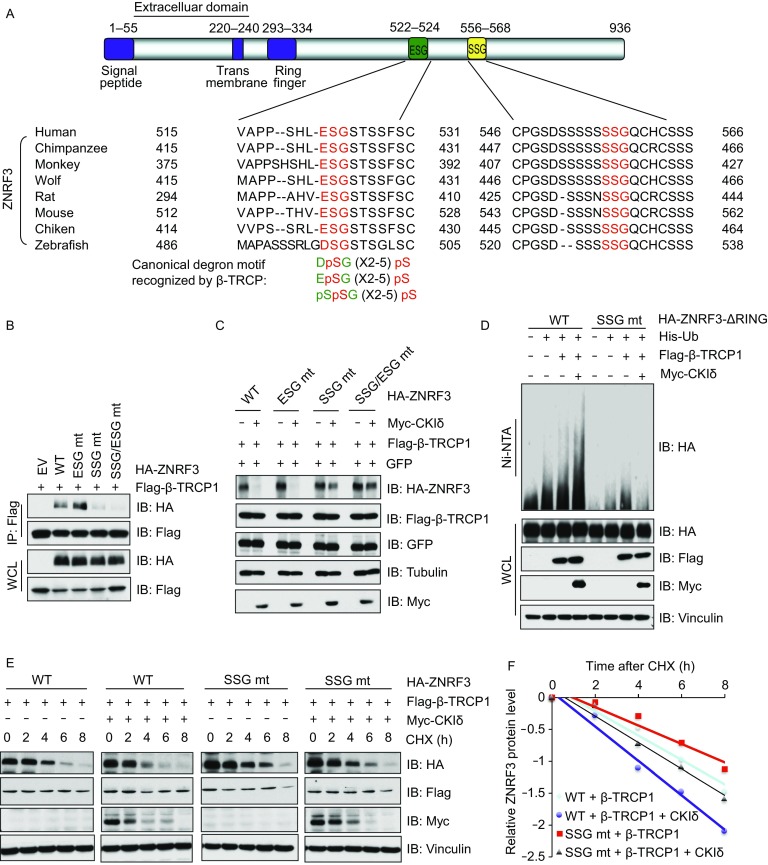
**β-TRCP promotes the degradation of ZNRF3 in a degron-dependent manner**. (A) A schematic illustration of the domain structure and putative β-TRCP-degron motifs in ZNRF3, as well as the sequence alignment of ZNRF3 among different species to illustrate evolutionary conservation of degrons. Where indicated, the canonical β-TRCP-degron motifs are shown. (B and C) IB analysis of IP and WCL derived from HeLa cells transfected with indicated constructs. (D) IB analysis of ubiquitin-products and WCL derived from HEK293 cells transfected with indicated constructs. Where indicated Nickel-beads were used to pull down His-tagged Ub proteins. (E and F) IB analysis of WCL derived from HeLa cells transfected with indicated constructs and treated with CHX (100 μmol/L) for indicated time points (E), the relative protein levels were quantified and plotted in (F)

It is well established that many F-box proteins recognize degron motifs within their target proteins, typically in combination with post-translational modification of the degron motifs such as phosphorylation, acetylation, methylation, or glycosylation (Shimizu et al., 2017; Westbrook et al., 2008). Upon scanning the protein sequences, we found putative evolutionary conserved β-TRCP degron motifs in ZNRF3 and its homolog protein RNF43 in the intracellular domain (Figs. 4A and S3A). To determine whether β-TRCP recognizes either ZNRF3 or RNF43 in a degron-dependent manner, we mutated the putative degron motifs (ESG and/or SSG in ZNRF3; DSG in RNF43). We observed that mutating SSG in ZNRF3 but not other putative degron motif in ZNRF3 or RNF43 could abolish the interaction with β-TRCP (Figs. 4B and S3B), indicating that β-TRCP interacted with ZNRF3 in a degron-dependent manner.

To further validate the potential role of the degron motif in ZNRF3 turnover, we co-expressed ZNRF3 harboring different degron mutations in the presence of CKIδ and β-TRCP. We found that the SSG degron deleted form of ZNRF3 was more resistant to CKIδ/β-TRCP-mediated degradation (Fig. 4C). Furthermore, β-TRCP1-induced ubiquitination of ZNRF3-ΔRing was enhanced by CKIδ, but ubiquitination of the SSG-deleted form of ZNRF3-ΔRing was not modulated by the combinational expression of CKIδ/β-TRCP (Fig. 4D). Next, we observed that the SSG-mutated form of ZNRF3 not only blocked β-TRCP-shortened ZNRF3 half-life, but also decreased CKIδ-mediated turnover of ZNRF3 (Figs. 4E, 4F, S4A, and S4B). To further confirm the degrons-mutated function of ZNRF3 in Wnt active conditions, we ectopically expressed WT and degron-mutated (SSG-mut) ZNRF3 in HeLa cells and treated with or without wnt3a proteins. We observed that active Wnt pathway could elevate β-catenin levels in ZNRF3 WT cells, however, the elevated β-catenin could be compromised by expressing SSG-mut ZNRF3 (Fig. S4C). As a result, the expression of downstream substrates of β-catenin, including Axin-2, Cyclin D1 and c-Myc were markedly decreased consistent with the level of β-catenin (Fig. S4C). All these findings indicate that the expression of degrons-mutated form of ZNRF3 could escape β-TRCP-mediated ubiquitination and degradation, in turn to antagonize the wnt3a-induced β-catenin and its downstream signals (Fig. S4C).

## Discussion

**Figure 5:**
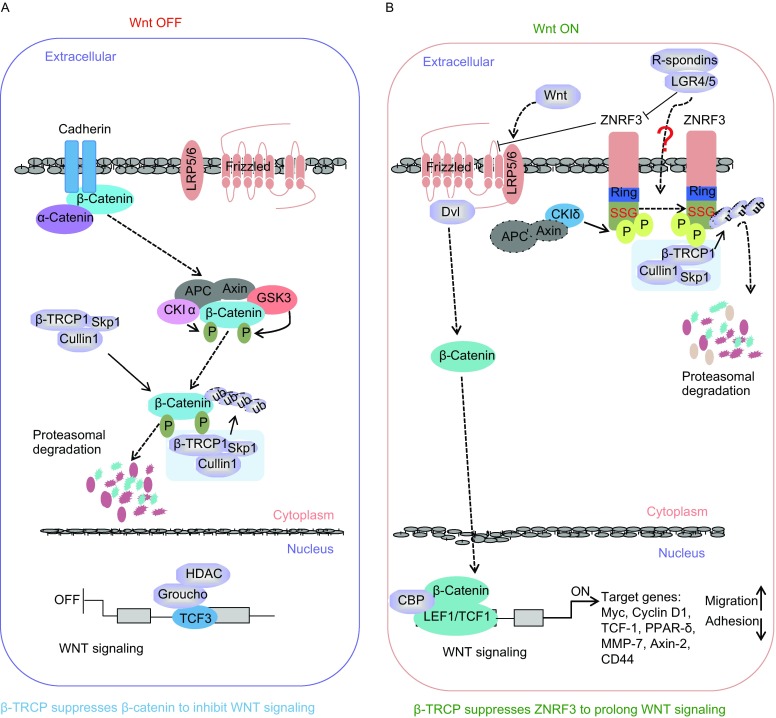
**Proposed models for the context-dependent roles of β-TRCP in regulating the Wnt signaling**. (A) Under Wnt off conditions, β-TRCP formed a degradation complex to recognize and degrade CKIα/GSK3-mediated phosphorylated form of β-catenin, to repress β-catenin targeted genes, and maintain Wnt in silent status. (B) Under Wnt on conditions, the activated FZD/LRP5/6 receptor destructs β-catenin degradation complex and releases β-catenin downstream targeted genes. Simultaneously, as a feedback regulator of Wnt pathway, ZNRF3 degrades FZD to alleviate Wnt signal. Meantime, β-TRCP1 could promote the degradation of CKI-mediated phosphorylated form of ZNRF3, to sustain the Wnt pathway

As the central mediator of the Wnt pathway, β-catenin is tightly controlled by the well characterized “destruction complex” including adaptor proteins, Axin and APC, kinases CKI and GSK3β, protein phosphatase 2A (PP2A), as well as E3-ubiquitin ligase β-TRCP (Rubinfeld et al., 1996; Kimelman and Xu, 2006). In detail, APC/Axin build a platform to facilitate β-catenin phosphorylation by CKI/GSK3, in turn the phosphorylated β-catenin is then recognized by β-TRCP and undergoes ubiquitination and degradation in the absence of Wnt stimuli (Hart et al., 1999; Behrens et al., 1998). Through this mechanism, β-TRCP displays a tumor suppressive role in Wnt pathway by targeting β-catenin (Fig. 5A). Here, we report that β-TRCP directly ubiquitinates and mediates the degradation of another E3 ligase ZNRF3 (Fig. 2). Since ZNRF3 plays a negative role in regulating the Wnt signaling pathway in Wnt on conditions (Hao et al., 2012), our findings reveal that β-TRCP plays an oncogenic role in the presence of Wnt stimuli by targeting ZNRF3 (Fig. 5B). β-TRCP has previously been shown to function as a tumor suppressor by targeting various oncoproteins such as β-catenin, CDC25A, FBXO5, IκB and DEP domain-containing mTOR-interacting protein (DEPTOR) (Guardavaccaro et al., 2003; Gao et al., 2011; Busino et al., 2003). β-TRCP mutations have been reported in various tumors indicating potential oncogenic functions (Fuchs et al., 2004). These reports and our findings provide a context (Wnt status) dependent manner by which β-TRCP functions as a tumor suppressor or oncogene in Wnt signaling by targeting distinct substrates.

In our present study, we not only found that ZNRF3 interacts with SCF^β-TRCP^ components including Cullin 1, Skp1, Rbx1 and β-TRCP (Figs. 1 and 2), but also observed that the interaction between ZNRF3 with β-TRCP occurs in a CKI-mediated phosphorylation-dependent manner (Fig. 3), which is similar to the regulation of β-catenin by β-TRCP. Turnover of β-catenin protein is tightly controlled by the destruction complex (APC/Axin/CKI/GSK3/β-TRCP) (Hart et al., 1999), in which mutation of either *APC* or *AXIN*, as well as inactivation of GSK3 can impair the degradation of β-catenin (Sparks et al., 1998; Rubinfeld et al., 1993), in turn these alterations directly link this pathway to hereditary diseases, including colon cancer (Morin et al., 1997; Lammi et al., 2004). Thus, whether ZNRF3 is controlled by the similar destruction complex as β-catenin (Fig. 5B), and in turn alterations of *APC*/*AXIN* modulate ZNRF3 turnover, merit further investigation.

The homolog of ZNRF3, RNF43, is induced and regulated in the Wnt pathway and both play similar functions to ubiquitinate and degrade Frizzled receptors (Koo et al., 2012). However, in our current study, although we have observed the interaction of RNF43 with β-TRCP with comparable level as ZNRF3 (Fig. S1C), the interaction of RNF43 was not impaired by F-box mutation of β-TRCP (S474A) (Fig. S1D), furthermore, the potential degron deletion could not block the interaction of RNF43 with β-TRCP (Fig. S3). Together, our findings suggest that RNF43 may not be an ubiquitin substrate of β-TRCP, at least in our experimental condition.

In summary, here we report that the β-catenin E3-ubiqutin ligase, β-TRCP, could undergo an oncogenic role by ubiquitinating and thereby targeting ZNRF3 for degradation, to maintain Wnt signaling in the presence of Wnt stimuli. Furthermore, recognition of ZNRF3 by β-TRCP is mediated through a CKI-phosphorylation- and degron-dependent manner. These findings not only identify a novel substrate for β-TRCP leading to oncogenic signaling, but also highlight the delicate context-dependent roles of β-TRCP in Wnt signaling.

## Materials and methods

### Cell culture and transfection

HEK293, HEK293T, HeLa, U2OS cells were obtained from ATCC and cultured in DMEM medium containing 10% FBS, 100 units of penicillin and 100 mg/mL streptomycin. Cell transfections were performed using Lipofectamine 2000. Packaging of lentiviral shRNA viruses, as well as subsequent infection of various cell lines was performed as previously described (Guo et al., 2016).

Wnt-3A cells (CRL-2647) and control L cells (CRL-2648) were kind gifts from Dr. Xi He and cultured according to the manufacturer’s instructions (ATCC).

### Antibodies and reagents

Mouse monoclonal anti-Myc-Tag (2276), rabbit monoclonal anti-Myc-Tag antibody (2278), anti-β-TRCP (4394), anti-Cyclin D1 (2978), anti-LRP6 (3395), rabbit polyclonal anti-Cullin 1 (4995) antibodies were purchased from Cell Signaling. Anti-HA antibody (SC-805), anti-Tubulin (SC-73242), anti-c-Myc (SC-40), anti-Axin-2 (SC-8570) and anti-p27 (SC-527) antibodies were purchased from Santa Cruz. Anti-ZNRF3 antibody (ab176449) was purchased from Abcam. Anti-GFP (8371-2) antibody was purchased from Clontech. Anti-β-catenin (AV100600), polyclonal anti-Flag antibody (F7425), monoclonal anti-Flag antibody (F-3165, clone M2), anti-Vinculin antibody (V-4505), peroxidase-conjugated anti-mouse secondary antibody (A-4416), peroxidase-conjugated anti-rabbit secondary antibody (A-4914), anti-HA agarose beads (A-2095) and anti-Flag agarose beads (A-2220) were purchased from Sigma. All antibodies were used in 1:1000 dilutions in 5% non-fat milk for Western blot. Proteasome inhibitor MG132 (S2619) and CKI inhibitor D4476 were obtained from Selleckchem. CHX (C4859) was purchased from Sigma; the neddylation inhibitor MLN4924 was a kind gift from Dr. William Kaelin (Dana-Farber cancer institute). λ-PPase (P0753S) was obtained from New England Biolabs.

### Plasmid construction

HA-ZNRF3 and HA-RNF43 constructs were obtained from Dr. Xi He (Harvard Medical School). Myc-Cullin 1, Myc-Cullin 2, Myc-Cullin 3, Myc-Cullin 4A, Myc-Cullin 5, shScramble, sh-Cullin 1, Myc-Rbx1, Myc-Skp1, pCDNA-GFP, Flag-β-TRCP, Flag-β-TRCP-R474A, shRNAs against β-TRCP1/β-TRCP2 and His-ubiquitin constructs were described previously (Shimizu et al., 2017). Different isoforms of CKI and CKII, as well as shRNAs against CKI were described previously (Shimizu et al., 2017). HA-ZNRF3-ΔRING and ZNRF3/RNF43 deletion degron mutants were generated with QuikChange Multi Site-Directed Mutagenesis Kit (Agilent) following the instructions.

### Immunoblot, immunoprecipitations (IP) and GST pull-down assay

Cells were lysed in EBC buffer (50 mmol/L Tris pH 7.5, 120 mmol/L NaCl, 0.5% NP-40) supplemented with protease inhibitors and phosphatase inhibitors (Complete Mini, Roche). The protein concentrations of whole cell lysates were measured using the Bio-Rad protein assay reagent. Equal amounts of whole cell lysates were resolved by SDS-PAGE and immunoblotted with indicated antibodies. For immunoprecipitation and GST pull-down analyses, 1 mg lysates were incubated with the indicated HA- or Flag-conjugated sepharose beads (Sigma) or 50% glutathione-sepharose slurry (GE) for 3–4 h at 4°C. The immuno-complexes were washed four times with NETN buffer (20 mmol/L Tris, pH 8.0, 150 mmol/L NaCl, 1 mmol/L EDTA and 0.5% NP-40) before subject to SDS-PAGE analysis.

### Protein degradation analysis and protein half-life assays

Cells cultured in 6-cm dishes were transfected with 0.1 μg Flag-ZNRF3, along with different concentration of β-TRCP or CKI. For half-life studies, 100 μg/mL CHX (Sigma-Aldrich) was added to the cells 36 h post transfection. At the indicated time points, cells were harvested and protein concentrations were measured. Total 60 μg of the indicated whole cell lysates were separated by SDS-PAGE and protein levels were measured by immunoblot analysis.

### *In vivo* ubiquitination assays

His-ubiquitin along with Flag-β-TRCP and ZNRF3 were transfected into cells. Thirty-six hours post transfection, cells were treated with MG132 (10 μmol/L) for overnight, and were then lysed in buffer A (6 mol/L guanidine-HCl, 0.1 mol/L Na_2_HPO_4_/NaH_2_PO_4_, and 10 mmol/L imidazole [pH 8.0]) and subjected to sonication. After centrifugation, supernatants were incubated with nickel-beads (Ni-NTA) (Qiagen) for 3 h at room temperature. The products were washed twice with buffer A, twice with buffer A/TI (1 volume buffer A and 3 volumes buffer TI), and one time with buffer TI (25 mmol/L Tris-HCl and 20 mmol/L imidazole [pH 6.8]). The pull-down proteins were resolved in 8% SDS-PAGE for immunoblot analysis.

## Electronic supplementary material

Below is the link to the electronic supplementary material.

Supplementary material 1 (PDF 453 kb)
