# Supplementary material for: SCFβ-TRCP E3 ubiquitin ligase targets the tumor suppressor ZNRF3 for ubiquitination and degradation
Source: Protein Cell. 2018 Mar 1;9(10):879–89. doi: 10.1007/s13238-018-0510-2 (PMC6160385; doi:10.1007/s13238-018-0510-2)
Supplement: Supplementary file 1 — Supplementary material 1 (PDF 453 kb) [file 13238_2018_510_MOESM1_ESM.pdf]

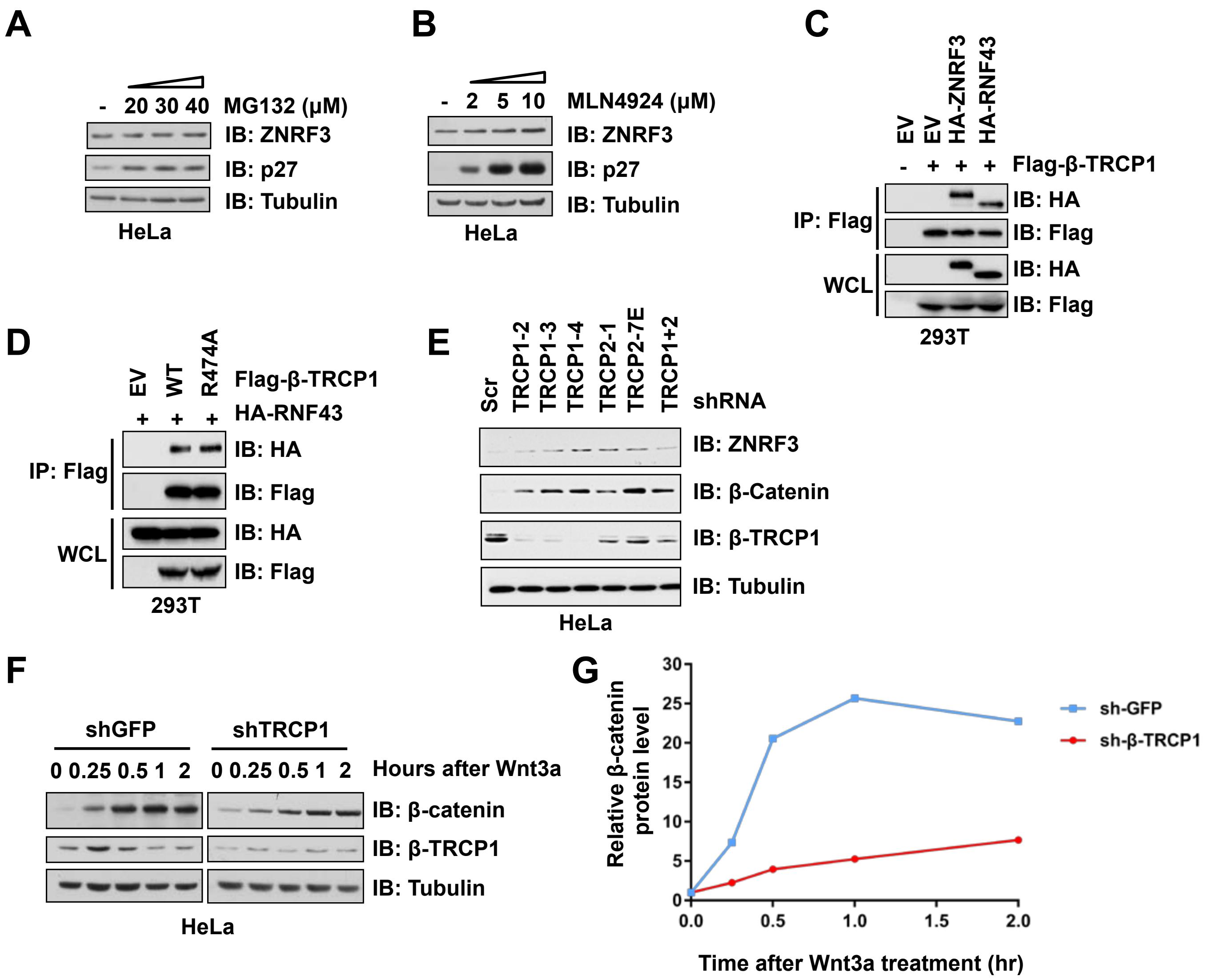

Figure S1. ZNRF3/RNF43 interacts with TRCP1

A

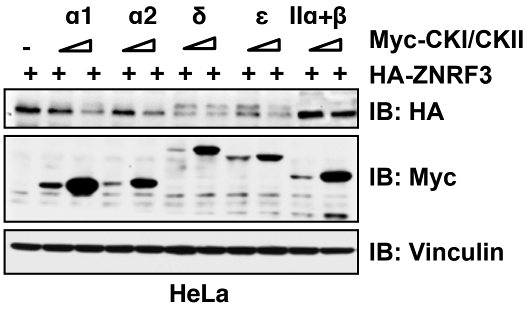

Figure S2. CKI, but not CKII, promotes the degradation of ZNRI

A

Canonical degron motif recognized by β-TRCP:

DpSG(X2-5)pS
EpSG(X2-5)pS
pSpSG(X2-5)pS

RNF43

Human	244	ATRRYQASCRQARGEWPDSGSSCSSAPVCAICLEEFSEGQELRVISCLHE	293
Chimpanzee	244	ATRRYQASCRQARGEWPDSGSSCSSAPVCAICLEEFSEGQELRVISCLHE	293
Monkey	244	ATRRYQASCRQAQGEWPDSGSSCSSAPVCAICLEEFSEGQELRVISCLHE	293
Cattle	244	ATRSYRAGCRGARKEWPDSGSSCSSAPVCAICLEEFSEGQELRVISCLHE	293
Wolf	244	ATRRYRASCKRARAEWPDSSSSCNSAPVCAICLEEFSEGQELRIISCLHE	293
Rat	244	ATRRYQASCRRARAEWPDSGSSSSSAPVCAICLEEFTEGQELRVISCLHE	293
Mouse	244	ATRRYQAGCRRARAEWPDSGSSCSSTPVCAICLEEFSEGQELRVISCLHE	293
Chicken	246	ATRRYQARCRQA--SWWDSASSCSSAPVCAICLEEFTEGQELRIISCSHE	293

B

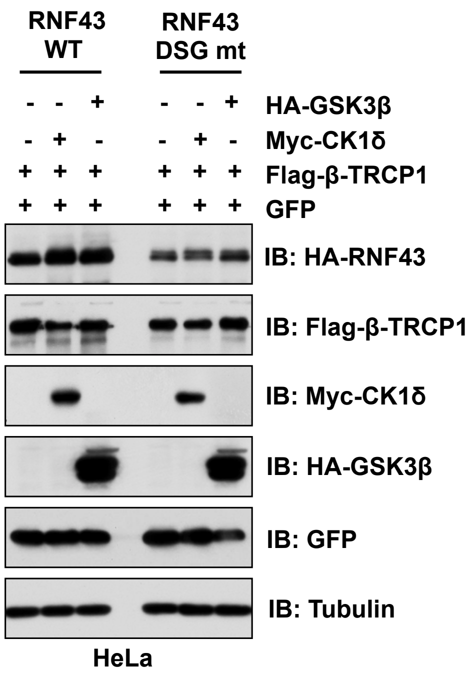

Figure S3. β-TRCP does not promote the degradation of RNF43

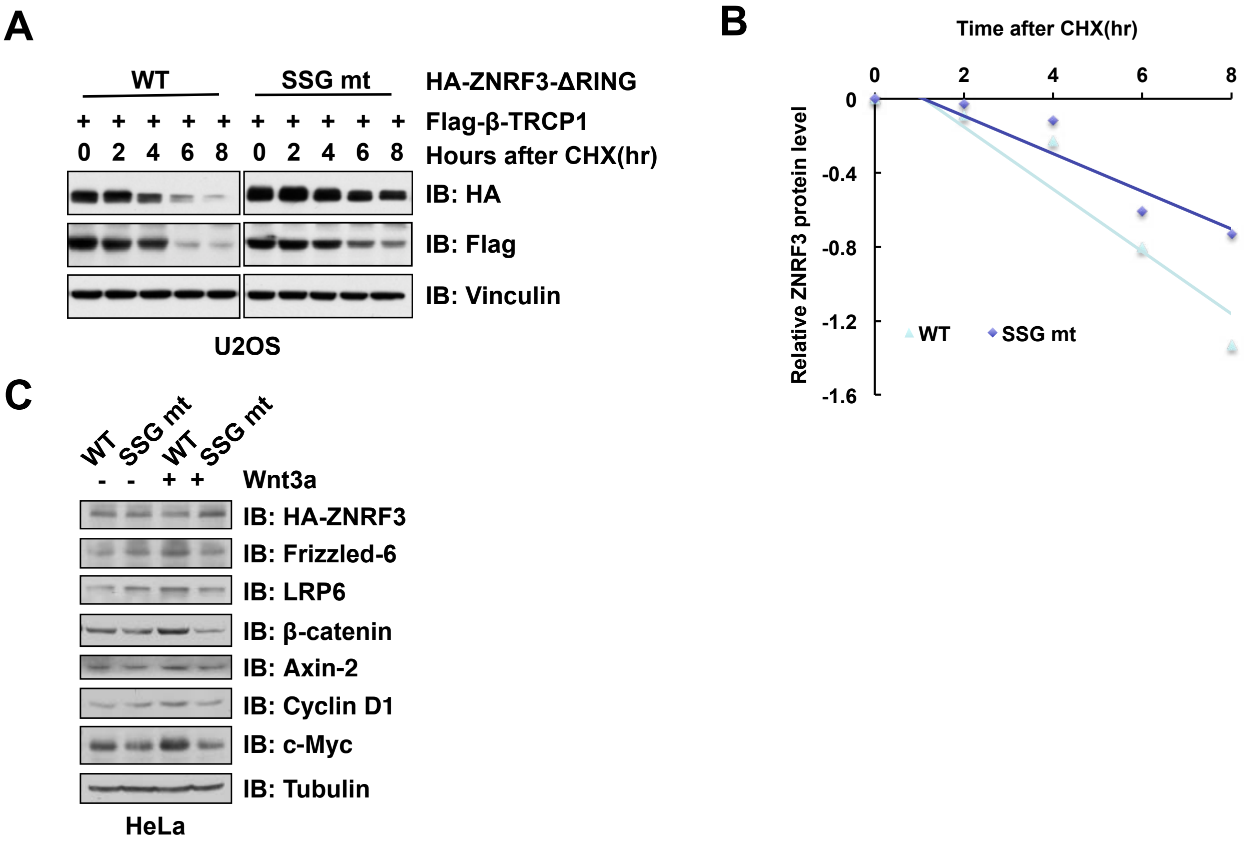

Figure S4. β-TRCP promotes the degradation of ZNRF3 in a degron dependent manner

SUPPLEMENTARY MATERIALS

Figure S1. ZNRF3/RNF43 interacts with TRCP1

(A-B) IB analysis of WCL derived from HeLa cells treated with MG132 or MLN4924 different does for 12 h before harvesting.

(C-D) IB analysis of IP and WCL derived from HEK293T cells transfected with indicated constructs and were treated with MG132 for 12 h before harvesting.

(**E**) IB analysis of WCL derived from HeLa cells infected with lentivirus encoding control (sh-Scr) or multiple independent shRNAs against *TRCP* (sh-TRCP). Infected cells were selected with 1 g/mL puromycin for 72 hr to eliminate non-infected cells before harvesting.

(**F-G**) HeLa cells were lentivirally infected with shRNA against *β-TRCP1* and selected with puromycin (1 μg/mL) for 5 days, and the resulting cells were stimulated with wnt3a proteins for different time point and subjected to IB analysis (F). The relative β-catenin protein levels were quantified with tubulin and normalized with time=0 in G.

Figure S2. CKI, but not CKII, promotes the degradation of ZNRF3

(A) IB analysis of IP and WCL derived from HeLa cells transfected with indicated constructs.

Figure S3. β-TRCP does not promote the degradation of RNF43

(**A**) A schematic illustration of the domain structures and putative β-TRCP-degron motifs in RNF43, as well as the sequence alignment with RNF43 from various species to illustrate the evolutionary conservation of this domain. Where indicated, the canonical β-TRCP-degron motifs are shown.

(B) IB analysis of IP and WCL derived from HeLa cells transfected with indicated constructs.

Figure S4. β-TRCP promotes the degradation of ZNRF3 in a degron dependent manner

(**A**-**B**) IB analysis of WCL derived from U2OS cells transfected with indicated constructs and treated with CHX (100 μM) for indicated time points (**A**), the relative proteins levels were quantified and plotted in (**B**).

(**C**) HeLa cells lentivirally infected with shRNA against ZNRF3 were infected with ZNRF3-WT or SSG-mut ZNRF3 retrovirus, and selected with hygromycin (200 μg/mL) for 5 days. The resulting cells were stimulated with or without wnt3a and subjected for IB analysis.
